# Letter-writing to the deceased among family caregivers of individuals living with dementia: exploring the role of technology-based interventions

**DOI:** 10.3389/fmed.2025.1576298

**Published:** 2025-04-07

**Authors:** Alexander Manevich

**Affiliations:** ^1^Department of Clinical Psychology of Adulthood and Aging, Ruppin Academic Center, Hefer Valley, Israel; ^2^International Laboratory for the Study of Loss, Bereavement and Human Resilience and the School of Psychological Sciences, University of Haifa, Haifa, Israel

**Keywords:** dementia caregivers, pre-death grief, expressive writing, letter-writing to the deceased, technology-based interventions, artificial intelligence

## Introduction

Dementia grief is the emotional, cognitive, and behavioral response of the primary caregiver to the series of losses associated with the unique coping process of cognitive decline and dementia (1). This response arises from several key factors: (a) the incongruence between the “psychological death” of the individual living with dementia and their physical death; (b) the prolonged and ambiguous nature of their medical condition; (c) the deterioration of communication abilities between the person living with dementia and their family members; and (d) the adverse changes in relationships, family roles, and the personal freedom of the primary caregiver (2).

This type of pre-death grief constitutes a significant risk factor for psychological distress and functional impairment among primary caregivers, which may necessitate professional intervention both before and after the death of the person living with dementia. Potential consequences of the caregiving role include depression, anxiety, burden, and prolonged grief disorder (PGD). For instance, a systematic review indicated that approximately 17% of family caregivers of individuals living with dementia met the diagnostic criteria for PGD before the patient's death, while the post-mortem prevalence ranges from 6 to 26% (3).

In fact, a recent meta-analysis and critical literature review including over 60,000 participants have examined various factors related to PGD (i.e., depression, violent and/or sudden death, low education and income levels, female gender, anxious attachment style, and the loss of a child or spouse), and identified pre-death grief as the most significant predictor of PGD (4). These findings underscore the need for early identification of risk factors and the provision of appropriate therapeutic interventions for primary caregivers.

Over the past decade, there has been an increase in research examining the effectiveness of psychotherapeutic interventions aimed at improving mental wellbeing and reducing pre-death grief among family members of individuals living with cognitive decline and dementia. However, this field remains in its early developmental stages, necessitating further investigation. Most existing interventions adopt cognitive-behavioral therapy (CBT) and problem-solving strategies (5–9). However, these approaches often do not fully address the multifaceted grief experience associated with the gradual loss of the relationship with the loved one living with cognitive decline and dementia.

Grief is not merely how individuals function or the distress they experience after a loss, but also how they reorganize their relationship with the deceased and their life without them. Neglecting the cognitive and emotional reconstruction related to the lost loved one may overlook a fundamental aspect of interpersonal loss, potentially resulting in interventions that do not fully address the needs of grieving individuals. In line with the Two-Track Model of Bereavement (10), it could be argued that most interventions have focused primarily on Track 1 of the model (biopsychosocial functioning) while largely disregarding the second track (mourning). Given this imbalance, it is unsurprising that the therapeutic effect sizes reported in meta-analyses of interventions for primary caregivers tend to be small to moderate at best (11).

Consequently, some researchers (12) have proposed adapting evidence-based interventions from the field of thanatology (the study of loss and grief), particularly those emphasizing the continuing bond with the deceased (13), and applying them to non-death related losses, such as dementia.

## Expressive writing and pre-death grief among primary caregivers of individuals living with dementia

In recent years, accumulating evidence has supported the effectiveness of creative arts therapies, such as music therapy, drama therapy, movement therapy, poetry, and writing in facilitating adaptive grieving processes (14). Expressive writing is a psychological intervention developed in the 1980s, wherein participants are instructed to write about a distressing or traumatic life event (typically for 15–20 min across three to five consecutive sessions). There are various ways to engage in expressive writing, either within a therapeutic context (individual or group), which allows for professional feedback, or through independent methods, such as personal journaling, blogging, etc. The choice of approach depends on the individual's preferences and the specific goals for using the technique. This method offers a safe space for individuals to freely express their emotions, thoughts, and experiences related to distressing events, thereby promoting psychological wellbeing (15).

Research supports the benefits of writing about traumatic and stressful events in improving both physical and psychological health across clinical and non-clinical populations (16). For example, meta-analyses examining the effectiveness of expressive writing among people coping with cancer have found that it can alleviate physical symptoms (17, 18). Another meta-analysis (19) reported its positive effects on post-traumatic stress disorder symptoms, post-traumatic growth, and overall quality of life.

Moreover, preliminary findings suggest that expressive writing may reduce anxiety and depression symptoms among family caregivers of individuals living with Parkinson's disease (20) and assist in meaning-making processes among family members of individuals living with dementia (21). However, despite the relevance of these studies, they do not directly address the grief and loss experienced by family caregivers.

Letter-writing to the deceased is a common therapeutic technique derived from the field of thanatology, based on the principles of expressive writing. This method allows mourners to maintain symbolic communication with the deceased significant-other and rework emotions associated with their loss (22). It is widely used in bereavement interventions, and various evidence-based treatment protocols for PGD incorporate this technique. However, thus far, research has yet to establish the unique contribution and standalone efficacy of letter writing as an independent intervention (23).

To date, only two pilot studies have applied letter-writing to the deceased techniques specifically to individuals experiencing non-death related losses (12, 24). Den Elzen et al. (12) included both participants grieving a death-related loss and those facing non-death losses (e.g., divorce, infertility, parenting a child with disabilities, and dementia). While promising findings were reported regarding the technique's effectiveness across both groups (12), it should be noted that the overall sample size was small (*N* = 20). Additionally, the non-death loss group was highly heterogeneous, necessitating a cautious interpretation of these results.

A recent study (24) presented two case studies in which letter writing was used specifically with adult children caring for a parent living with dementia. The researchers concluded by emphasizing the potential of this approach to facilitate benevolent continuing bonds (13) and adaptive grieving while the loved one is still alive.

## Discussion

Dementia grief is the emotional, cognitive, and behavioral response of primary caregivers to the unique losses associated with the disease. This form of pre-death grief is a significant risk factor for psychological distress and functional impairment, both before and after the death of the affected individual.

Despite a growing body of research on dementia grief among family caregivers, this field remains in its infancy. Most intervention studies have been based on CBT approaches, which are effective in alleviating distress; however, they still require further development to fully address the unique aspects of pre-death grief. While addressing the biopsychosocial functioning of the caregiver is crucial (e.g., depression, anxiety, caregiver burden), it is equally important to consider the continuing bond with the significant other, which remains central to the grieving process. The absence of professional attention to this aspect risks partial or even inaccurate assessment, potentially leading to suboptimal treatment.

Letter writing to the deceased is an expressive writing technique widely used in bereavement therapy, allowing individuals to sustain a dialogue with their “lost” loved one and process their relationship. This technique is notable for its simplicity, accessibility, and practicality, as it requires minimal financial resources and is adaptable across diverse populations (both clinical and non-clinical). Additionally, recent evidence suggests its potential utility in addressing pre-death grief among family caregivers of individuals with dementia, highlighting the need for further research in this area.

In addition to the advantages described above, the integration of technological means—such as the development of a dedicated application—both within clinical and community settings, may enhance the quality of professional services provided to the families of individuals living with dementia. Specifically, it could contribute to advancing the potential embedded in the letter-writing technique within this population, whether as part of a formal therapeutic framework or independently. It should be noted that the aim of the present article is to lay the groundwork and propose a general framework for implementing the discussed technique through digital means, highlighting the inherent benefits of such an approach, without being constrained by or dependent on any specific protocol.

Provided that appropriate measures are taken to ensure privacy and secure sensitive information on digital platforms, the use of technology may increase the accessibility of interventions to broader populations, including those who avoid receiving treatment or face difficulties attending in-person therapy sessions. Moreover, it may reduce dropout rates from treatment, offer flexibility and convenience (in terms of location, scheduling, and writing format), and lower economic costs. Additionally, the use of technology could facilitate the preservation of data for ongoing monitoring and tracking of therapeutic progress through reliable and validated assessment tools [e.g., MM-CGI, CGS (25)], along with the provision of professional, personalized feedback via remote access.

Recent developments in artificial intelligence (AI) technology open new possibilities for mental health support systems, but also introduce significant professional and ethical dilemmas. Therefore, both the opportunities and risks associated with its integration into assessment and treatment processes, particularly in responses to grief among the families of individuals living with dementia, must be carefully and thoughtfully examined. It should be emphasized that this integration is intended to assist human professionals in delivering psychosocial care—not replace them.

One of many examples of this is the potential use of AI in letter-writing to the deceased (e.g., text analysis that provides insights into the emotional state of the writer, creation of personalized guidelines, and even simulation of dialogues with the deceased to assist in processing loss), which illustrates the broad range of possibilities available to the mental health field.

In conclusion, it must be emphasized that humanity undergoing a revolution, which, alongside the concerns it raises, has become an inseparable part of both the present and future reality. Therefore, the professional community must prepare for the responsible and informed integration of this technology as a means to enrich and support therapeutic processes, while rigorously maintaining professional ethics and the highest standards of the care provided.
